# Nitrous Oxide for Dental Anæsthesia

**Published:** 1866-06

**Authors:** A. W. Sprague

**Affiliations:** Boston, Mass.


### NITROUS OXIDE FOR DENTAL ANÆSTHESIA.

BY DR. A. W. SPRAGUE, BOSTON, MASS.

That nitrous oxide is and has been successfully employed as an anæsthetic, no intelligent person will deny. Is its use practical? This is the question. After four years of practical systematic experimenting on the chemical, physiological and anæsthetic properties of this gas, I am thoroughly convinced that, with proper appliances and skill, it is cheapest, most healthy, and most convenient agent for brief anæsthesia, yet devised.

Opinions upon all practical subjects vary with experience. One dentist with a whiskey cask for a washer and holder of the gas, and a filthy three gallon inhaling bag and wooden mouth-piece for administering it, writes out an elaborate opinion, stating that nitrous oxide is not only very expensive—will not keep—but is attended with unpleasant results in many cases where it has been administered. Another says he has used a "very convenient" arrangement of *three one-half gallon* washers, (?), and a rubber bag for preparing the gas as wanted, but finds the physiological effects in many cases decidedly unfavorable; the patient in some cases partially chooking, with a subsequent tendency to cough. Another, a very learned chemist, who has read Sir Humphrey Davy's partial researches, with a very rude apparatus, but has never made or used it himself, thinks nitrous oxide not safe,—sees funeral trains, open graves, and untold horrors, as the necessary concomitant of anæsthesia by this gas. A fourth, who has prepared the gas at a moderate heat, washed it thoroughly, confined it in a proper receiver over water, and given it pure from the same, tells us, that of some three thousand cases where he has employed it for anæsthesia, (in some protracted to twelve and fifteen minutes), he has never had an unfavorable result, and believes nitrous oxide the safest and cheapest anesthetic he has ever used. Thus, opinions of this agent, vary according to the means and skill employed.

With improper appliances no agent suffers more by attempts to use. The process of preparation is a chemical process, and for economy and safety, must be conducted on chemical principles. The inhaling must be in accordance with physiological laws. Outrage of either these and nature will rebuke the same.

No anæsthetic and therapeutic has struggled against such odds of ignorance and abuse, and yet lived.

The great mass know little of this agent except by the result of inhaling and what they are told by the profession. Many who advise know only a few mechanical details. One operator makes a crude, semi-poisonous compound, to which he perhaps adds, in administering it, the horrors of partial asphyxia. To nitrous oxide, not to the admixed, chlorine, carbonic acid, etc., given it by the operator, is charged the headache, strangulation and cough which may result.

An itinerant lecturer upon nitrous oxide, recently told us, that once when he had gathered an audience to witness the effects of gas, his rude apparatus failing him, he retired to a side room, filled his bag with air, gave it, produced the desired delirium, satisfied his audience, and heard no serious results from the deception except *a little subsequent headache* to the victims.

For anæsthesia, nitrous oxide should be made from the purest nitrate of amonia, the gas thoroughly washed by *straining* through several separate glass strainers, and then kept in a proper holder over water. In this way it may be retained good for use for six weeks. The inhalation should be direct from the gas holder by means of a proper valve inhaler, so that no carbonic acid or respired breath can be re-inhaled. In this way perfect anæsthesia, *with less gas*, may be produced, while the results will be far more satisfactory, than where cleanliness is outraged and the patient half asphyxiated by breathing the excretions from his own and other lungs in a small confined bag,—we state facts. Some forty dentists of Eastern Massachusetts have adopted the pure gas process, and all concur in the above opinion we have expressed.

